# Metabolic Complications of Bypass Surgery for Morbid Obesity

**DOI:** 10.4137/ccrep.s3226

**Published:** 2009-09-18

**Authors:** S. Richard-Devantoy, J.B. Garré, B. Gohier

**Affiliations:** 1 Département de Psychiatrie et Psychologie médicale, Centre Hospitalier Universitaire d’Angers, 4 rue Larrey, Angers, France.; 2 UPRES EA 2646, Université d’Angers, UNAM, Angers, France.

**Keywords:** bariatric surgery, Wernicke’s encephalopathy, morbid obesity, metabolic complications, prevention

## Abstract

Postoperative complications resulting from bariatric surgery can lead to severe vitamin-deficiency states. A patient who underwent bariatric bypass surgery and later developed Wernicke’s encephalopathy prompted us to present her interesting case history for discussion. Although bariatric surgery is known to be a risk factor for Wernicke’s encephalopathy, this diagnosis is only rarely evoked in the postoperative course. We recommend that the occurrence of digestive, psychiatric or neurological symptoms after bariatric surgery should suggest a thiamine deficiency that must be promptly assessed. Without waiting for the results, thiamine supplementation should be initiated.

## Introduction

Support for the current epidemic of morbid obesity affecting industrialized countries requires a multidisciplinary approach in which surgery is a growing (Body Mass Index (B.M.I.) >40 or B.M.I. > 35 and comorbidities).^1^ This type of treatment, which should be reserved for selected patients, allows for weight loss and improved cardiovascular satellites comorbidities of obesity such as diabetes, hypertension and dyslipidemia.^2^ Patients suffering from morbid obesity often have nutritional deficiencies, including fat-soluble vitamins, folate and zinc.^3^ After bariatric surgery, these gaps are widening. Others may appear, especially because of the restriction of dietary intake in the gastric reduction surgery, and malabsorption induced in type interventions bypass. The latter lead to greater weight loss but also to exhibit more severe deficiencies. Besides a protein undernutrition with decreased lean mass, an iron deficiency anemia from deficiency in folate or vitamin B 12 can also observe the neurological manifestations such as encephalopathy of Gayet-Wernicke. Wernicke’s encephalopathy results from vitamin B1 (thiamine) deficiency and has been associated with alcoholism, gastric cancer, pyloric obstruction, hyperemesis gravidarum, and prolonged parenteral feeding. Bariatric surgery appears to be another emerging condition that may lead to Wernicke’s encephalopathy. The average rates of early postoperative deaths were 0.1%, 0.35% and 0.5% respectively for gastric adjustable, the bilio-pancreatic diversion and gastric bypass. The pulmonary embolism was the most common etiology found, up to 70% of the vertical gastroplasty size poses and the order of 50% for the other two techniques.^1^ The diagnosis of encephalopathy of Gayet-Wernicke is generally referred to the history of bariatric surgery: vertical gastroplasty size poses,^4^ gastric ring,^5^ and most often gastric bypass.^6^

## Observation

In 2006, Mrs. K., a 34-year-old, underwent gastric bypass surgery after failed gastroplasty at the age of 18 and persisting morbid obesity (weight: 109 kg; height 1.65 m; body mass index: 40 kg/m^2^). Following surgery, the patient developed restrictive anorexia nervosa characterized by a voluntary refusal to eat, potomania (excess intake of water and coffee), a fear of weight gain, and a wish to accelerate weight loss. Within 3 months, she lost 30 kg or 27% of her initial weight. Given this self-imposed dietary restriction, 2 months after bypass surgery, Mrs. K., suddenly presented spontaneous nausea with vomiting along with a neurological episode characterized by feelings of vertigo, atactic walk, an intense rotatory sensation and a vertical-inferior rotary nystagmus. Laboratory screening did not reveal any abnormalities (blood cell count, blood ionogram as well as hepatic, renal and pancreatic functions). Investigations including a lombar punction, an electroencephalogramme and magnetic resonance imaging did not lead to any conclusive diagnosis. Vitamin levels (vitamins A, E, and B12, erythrocyte transketolase activity, serum and erythrocyte folates) were normal and all serologies were negative (Lyme disease, HIV, syphilis, CMV, herpes virus, EBV, hepatitis B and C, Yersinia, and Campylobacter jejuni). Immunological screening was normal (native anti-DNA antibodies, soluble nuclear antigens, anti-mitochondria antibodies, rheumatoid factor, complement, cold agglutinines, and ANCA). The diagnostic hypothesis proposed at first was that of acute polyradiculoneuritis as seen in Miller-Fisher syndrome. As the diagnosis of Wernicke’s encephalopathy was not initially evoked, thiamine concentrations were not measured. Mrs. K., who did not take any postoperative vitamins, was not immediately prescribed vitamin B1 supplementation.

Towards the end of 2007, Mrs. K., was admitted to the Angers University Hospital for a check-up of residual neurological problems. At that time, being 1.65 m tall, she weighted 52 kg, which corresponded to a BMI of 19 Kg/m^2^. The clinical picture was characterized as follows: major ataxia, only slightly aggravated by eye closure, a cerebellar kinetic syndrome predominant on the right side along with a discret hypoesthesia of the right hemibody involving the face without motor deficiency, abolition of osteotendineous reflexes as well as a cerebellar-like dysarthria. While the patient was well-oriented in space and time and was not in any state of confusion, she presented an anterograde and partial retrograde amnesia associated with signs of frontal lobe dysfunction, such as euphoria, reduced verbal fluency, and lack of words. The frontal assessment battery (FAB) which assesses frontal lobe function was evaluated at 14/18. The clinical symptoms evocative of cerebellar dysfunction associated with an anterograd mnesic disorder and a normal electromyogram reinforced the hypothesis of a sequella to Wernicke’s encephalopathy.

## Discussion

This is the only case observed within the Loire region of France. It should be noted that the patient was not followed by the Angers network for surgical treatment of morbid obesity.

In response to a steep increase in morbid obesity, new therapeutic options have been developed. Among these, bariatric surgery is one of the most effective methods for the long-term management of obesity and its complications.^7^ Provided that contraindications are respected, the method appears to be safe with only 0.5% to 3% of short and long-term complications and a mortality rate estimated at 0.5% in France.^1^ In contrast, current bariatric studies report a 20% in-hospital complication rate in United-States.^8^ Enconisa et al found a significantly higher complication rate over the six months after surgery, resulting in costly readmissions and emergency room visits.^8^ In addition to the complications related to the surgery such as stenoses of gastrojejunal anastomosis, internal hernias, ulcers and disruption of the suture lines, several research teams have highlighted the occurrence of nutritional complications, such as malnutrition due to protein deficiency, hypophosphatemia, and Wernicke’s syndrome. This latter complication is particularly serious because of the sequellae that it may induce in the case of an erroneous diagnosis. Of note, thiamine’s primary absorption occurs in the jejunum, which is partly by-passed by this type of surgery.

Yet Wernicke’s syndrome diagnosis is only rarely evoked although gastric bypass surgery is known to be a risk factor for this condition. Indeed, all types of bariatric surgery may lead to this complication and several predisposing risk factors have been identified, i.e. significant initial weight loss and vomiting.^9^,^10^ Intravenous glucose administration without thiamine was a risk factor in 18% cases of the literature.^10^ The vitamin B1 deficiency after bariatric surgery resulted in a decrease in acid production by the gastric pouch, a reduction in food intake and repeated episodes of vomiting. From a neurological perspective, Wernicke’s encephalopathy is associated with a Korsakoff syndrome. Typically, encephalopathy occurs within 4 to 12 weeks after surgery. Atypical neurological pictures are common and often misleading.^11^ Optic neuropathy, papilledema, deafness, seizures, asterixis, weakness, and sensory and motor neuropathy were also reported.^12^ Brain magnetic resonance imaging identified lesions characteristic of WE 47%.^10^ Characteristic radiographic findings were hyperintense signals in the periaqueductal gray area and dorsal medial nucleus of the thalamus; radiographs were normal in 15 patients.^12^ Although MRI has been reported to have high specificity (93%), its sensitivity is low (53%).^13^ Vitamin measurements are often flawed. For the reported cases, plasma thiamine levels and erythrocyte thiamine transketolase activity were normal in one-third of cases.^12^ The diagnosis is often made too late, based *a posteriori* on a bundle of arguments. Incomplete recovery was observed in 41 cases (49%); memory deficits and gait difficulties were frequent sequela.^10^

This observation illustrates the risks of vitamin deficiency and the necessity for its prevention following bariatric surgery. The few cases reported in the literature along with our observation allow us to put forth a simple but indispensable prevention strategy to reduce the risk of Wernicke’s encephalopathy. A preoperative assessment of thiamine levels does not appear to be pertinent. However, the pre-operative check-up should systematically include a screening for eating disorders, and the surgeons need to ascertain the patient’s compliance to treatment.

In the postoperative period after any type of bariatric surgery, the occurrence of digestive symptoms (anorexia, vomiting or significant weight loss), psychiatric symptoms (irritability or depression) or neurological symptoms should suggest a thiamine deficiency that must be promptly assessed. Considering the gravity of the sequellae in the case of delayed treatment, and without waiting for the results, vitamin supplementation must be initiated. And lastly, as thiamine is necessary for carbohydrate metabolism, glucose infusion after bariatric surgery always calls for thiamine supplementation.

According to the French recommendations, a very strict clinical, dietary and metabolic follow-up as well as vitamin monitoring at regular intervals must accompany all bariatric surgical interventions.^1^,^14^ This is necessary for the surgeons to ascertain that the patients are complying with proper hygienic and dietary measures and are taking their multivitamin supplementation, which may be adjusted as necessary. This complication is exceptional in France because the indication for surgery is taken by a multi-disciplinary staff after following the patient for an entire year. The post-operative follow-up continues in the long-term involving all the relevant parties (endocrinological nutritionist, general practitioner, surgeon, dietician, and psychiatrist). This highlights the interest of a network of professionals involved in the care of patients undergoing bariatric surgery. Wernicke encephalopathy after bariatric surgery usually occurs between 4 and 12 weeks postoperatively, especially in young women with vomiting. Atypical neurologic features are common. The diagnosis is mainly clinical, because radiographic findings are normal in some patients. Prospective studies to determine the prevalence of this problem and protocols for preventive thiamine supplementation need evaluation.
